# Oral Administration of *Clostridium butyricum* Alleviates High-Fat Diet-Induced Obesity in Mice by Modulating Gut *Akkermansia muciniphila* Abundance via Direct Growth Promotion

**DOI:** 10.4014/jmb.2509.09005

**Published:** 2025-12-09

**Authors:** Xinrui Dong, Li Lin, Lu Liu, Long Chen, Nan Shang, Ran Wang, Ming Zhang, Hao Zhang, Yanling Hao, Zhengyuan Zhai, Liang Zhao

**Affiliations:** 1 College of Food Science and Nutritional Engineering, China Agricultural University, Beijing 100083, P.R. China; 2 College of Engineering, China Agriculture University, 100083, P.R. China; 3 Key Laboratory of Functional Dairy, Department of Nutrition and Health, China Agricultural University, Beijing 100193, P.R. China; 4 School of Food and Health, Beijing Technology and Business University, Beijing 100048, P.R. China; 5 Research Center for Probiotics, China Agricultural University, Beijing 101299, P.R. China

**Keywords:** *Clostridium butyricum*, *Akkermansia muciniphila*, obesity, intestinal microbiota

## Abstract

This study investigated the anti-obesity effects of *Clostridium butyricum* (CLB) and the underlying mechanisms through gut microbiota modulation. High-fat diet (HFD)-fed C57BL/6 mice were orally administered CLB (1 × 10^10^ CFU/kg/day) for 8 weeks. CLB intervention reduced body weight gain by 13.20% and decreased fat mass (21.10 ± 2.24% vs. HFD: 23.39 ± 2.34%, *P* < 0.05), while partially restoring fecal butyrate levels (11.73 ± 4.99 vs. HFD: 7.27 ± 3.40 μg/g, *P* < 0.05). 16S rRNA gene sequencing revealed CLB selectively increased the abundance of *Akkermansia*, which was depleted in HFD mice. *In vitro*, butyrate, CLB lysate, and culture supernatant significantly enhanced the growth of *Akkermansia muciniphila* (AKK), indicating microbial cross-feeding. These findings demonstrate CLB alleviates obesity by restoring the abundance of AKK, partly through the production of metabolites that promote the proliferation of AKK, offering novel insights into probiotic-driven microbiota modulation for metabolic health.

## Introduction

Obesity has emerged as a pivotal global health challenge, with escalating prevalence rates. According to the World Health Organization (WHO), approximately 43% of adults worldwide were overweight in 2022, including 34.8% and 14.1% of Chinese adults categorized as overweight and obese, respectively. Obesity-associated complications, such as cardiovascular diseases, type 2 diabetes, and nonalcoholic fatty liver disease, contribute to over 4 million annual deaths globally, underscoring the urgency for effective interventions [1, 2].

The etiology of obesity involves complex interactions among genetic predisposition, environmental factors, and behavioral patterns. Notably, diets rich in fats and sugars but deficient in fiber disrupt energy homeostasis, leading to adipose accumulation [3, 4]. Emerging evidence highlights gut microbiota dysbiosis as a critical mediator of metabolic dysfunction [5, 6]. High-fat diets (HFDs) reduce microbial diversity, diminish beneficial taxa (*e.g.*, *Akkermansia muciniphila* and *Bifidobacterium*), and increase the abundance of Gram-negative bacteria, thereby increasing lipopolysaccharide (LPS) levels and reducing short-chain fatty acid (SCFA) production. These alterations impair intestinal barrier integrity, facilitating systemic LPS translocation, chronic inflammation, and insulin resistance, which collectively exacerbate obesity [7-9].

Probiotics and prebiotic interventions have demonstrated potential in mitigating obesity through the modulation of intestinal microbiota dysbiosis [10]. The specific effects of these interventions are believed to be due to their ability to produce SCFAs, which play a crucial role in energy homeostasis, immune modulation, and gut barrier function [11-13]. *Clostridium butyricum*, a spore-forming anaerobic bacterium, has shown efficacy in modulating gut microbiota composition, fostering a balanced microbial ecosystem that may be more resistant to dysbiosis and metabolic disturbances [14, 15]. Studies suggest that certain strains of *C. butyricum* can improve diet-induced obesity and related symptoms, such as insulin resistance and nonalcoholic fatty liver disease [16]. *C. butyricum* is thought to exert its beneficial effects through the production of SCFAs, particularly butyrate, which serves as an energy source for colonocytes and enhances the integrity of the gut barrier [17]. This may reduce the translocation of LPS and other inflammatory agents, mitigating the low-grade inflammation associated with obesity [18]. However, the impact of *C. butyricum* treatment on the gut microbiota's diversity or composition is an area of ongoing research. Previous studies have shown that *C. butyricum* can decrease the elevated abundance of certain bacterial groups (*Muribaculaceae* and *Enterococcus*) induced by a HFD in a strain-specific manner [19, 20]. However, its mechanisms of action, especially its influence on the mucin-degrading bacterium *A. muciniphila* negatively associated with obesity, remain underexplored.

This study investigates the anti-obesity effects of CLB in HFD-fed mice, focusing on its ability to restore AKK abundance. We hypothesize that CLB alleviates obesity not only via butyrate-mediated metabolic improvements but also through direct modulation of AKK proliferation. Using 16S rRNA sequencing and *in vitro* co-culture experiments, we elucidate the interplay between CLB, AKK, and host metabolism, providing novel insights into probiotic-driven microbiota modulation for obesity management.

## Materials and Methods

### Bacteria Strains and Cultures

*C. butyricum* (CLB, CGMCC 1.5205) was obtained from the China General Microbiological Culture Collection Center (CGMCC). *A. muciniphila* (AKK, ATCC BAA-835) was purchased from the American Type Culture Collection (ATCC). CLB and AKK were cultured anaerobically at 37°C in Reinforced Clostridium Medium and Brain Heart Infusion Broth (supplemented with 0.05% w/v cysteine hydrochloride), respectively. Bacteria were cultured for 18 h and cells were harvested by centrifugation. Cells were washed and resuspended in sterile saline. The viable count of the suspension was determined by plate counting method.

### Mice and Experiment Design

Five-week-old male C57BL/6 mice were acquired from Vital River Laboratory Animal Technology Co. Ltd.(China). All mice were maintained in a specific-pathogen-free environment on a 12-h light/dark cycle at 22 ± 2°C, 55 ± 15% relative humidity. All mice were allowed access to a standard chow diet and tap water *ad libitum*. The animal experiments were carried out in accordance with the recommendations of Animal Management Regulations, Ministry of Science and Technology of the People’s Republic of China. The protocol was approved by the Ethical Committee of Experimental Animal Care of China Agricultural University (No. KY180017).

After 1 week of acclimatization, 13 mice were randomly selected and fed a normal chow diet as a control group (Control), while the remaining mice were fed a HFD to induce obesity. The diet composition was shown in Table S1, and all diets were purchased from Beijing Huafukang Biotech Co., Ltd. (China). After 8 weeks of HFD feeding, the obesity model was considered successful when mice gained 15% more weight than that of the control group. Obese mice were randomly divided into two groups: HFD model group (HFD, *n* =8) and *C. butyricum* treatment group (HFD + CLB, *n* = 10). The mice in the control group and HFD group were intragastrically administered saline daily, and the HFD + CLB group was intragastrically administered CLB at 1.0 × 10^10^ CFU/kg body weight (BW) per day for 8 weeks.

### Obesity-Related Index Properties and Sample Collection

During the experiment, the body weights of mice were monitored weekly and the food amount was recorded weekly to calculate food intake. After the 8-week treatment period, all mice were fasted for 12 h, then they were anaesthetized with an intrabdominal injection of pentobarbital sodium. The body fat was measured using a Body Composition Analyzer MiniQMR23-060H-I (Shanghai Niumag Corporation, China). Subsequently, the mice were sacrificed by cervical dislocation. The intact liver and white adipose tissues (epididymis and retroperitoneal) were collected and weighed at the time of dissection. The adipose tissues were fixed by formalin and the paraffin blocks were cut into 4-μm sections. The sections were stained with hematoxylin and eosin (H&E) for histological examination. The fecal samples were collected 2 days before sacrifice for analysis of SCFAs and gut microbiota, as previously described.

### Measurement of Fecal SCFA Levels

The SCFAs were extracted from fecal samples as previously described [21]. Under low-temperature conditions, mouse fecal samples were suspended in ultrapure water (1 mg feces per 5 μl ultrapure water), vortexed thoroughly, and subsequently sonicated for 5 min. Hydrochloric acid solution (5 M) was then added to adjust the pH to approximately 2.5. The suspension was centrifuged at 12,000 × *g* for 15 min at 4°C, after which the supernatant was collected and filtered through a 0.22 μm membrane. The levels of acetic acid, propionic acid, and butyric acid were quantified by a gas chromatography system (GC 7890A, Agilent, USA) equipped with a flame ionization detector and an HP-FFAP chromatographic column (25 m, 0.32 mm, 0.5 μm) (Agilent, USA). The detector temperature was set at 280°C, with a hydrogen flow rate of 40 mL/min, an air flow rate of 400 mL/min, and a nitrogen (make-up gas) flow rate of 30 mL/min. The program was set as below: maintained at 50°C for 3 min, increased by 5°C/min to 140°C, kept at 140°C for 1 min, increased by 30°C/min to 240°C, and held at 240°C for 3 min. The injector temperature was set at 270°C, with a manual injection volume of 1 μl in split mode. High-purity nitrogen (≥99.999%) was used as the carrier gas at a flow rate of 1.2 mL/min, with a split ratio of 10:1. The peak area ratio of the target SCFA in the sample compared to that of internal standard (5 mM heptanoic acid) was measured, and then it was corrected by a relative correction factor. The corrected peak area ratio was used to calculate the concentration of the SCFA.

### Analysis of Intestinal Microbiota by 16S rRNA Sequencing

To analyze the microbial composition, fecal samples were analyzed by sequencing the V3-V4 region of the 16S rRNA gene. Fecal microbial DNA from each fecal sample was extracted with the phenol–chloroform method. Purification and quantification of PCR amplicons were performed according to standard procedures. Purified amplicons were pooled in equimolar concentrations and subjected to paired-end sequencing (2 × 250) on an Illumina MiSeq platform (Illumina, Inc., USA). The raw fastq files were demultiplexed and quality-filtered by QIIME (version 1.1.9) and then all effective reads from each sample were clustered into operational taxonomic units (OTUs) on the basis of 97% sequence similarity. The OTUs with proportional abundances of at least 0.1% in at least three samples were retained for downstream analysis. The taxonomy of each 16S rRNA gene sequence was analyzed by RDP Classifier against the SILVA v115 16S rRNA database [22].

The Sobs and Chao diversity indices of each sample were calculated based on OTUs in QIIME to compare gut microbial species richness in different groups. A principal coordinate analysis (PCoA) based on the weighted UniFrac distances of the OTUs was performed in R (vegan package). The relative abundances of the different genera in each sample were calculated and compared between groups using PERMANOVA. Heatmaps were generated in R using the pheatmap package.

To determine the intestinal bacteria involved in CLB-induced alleviation of obesity, we firstly retained the genera of ≥ 1% average abundance in all samples, which was identified as abundant genera. The potential obesity-related genera were obtained based on the significant differences in abundance between the HFD and Control groups (*P* < 0.1). Then, the changes of these genera were assessed between HFD and HFD + CLB groups (*P* < 0.1). Correspondingly, the obesity-enriched genera that were downregulated by CLB or obesity-reduced genera that were upregulated by CLB were considered potential obesity-related bacteria regulated by CLB (Table S2).

### Analyze the Impact of CLB on Growth of AKK *In Vitro*

To elucidate the mechanism by which *C. butyricum* affects the abundance of obesity-related bacterial targets in gut, two *in vitro* culture experiments were conducted to investigate the direct effects of CLB bacteria or metabolites on *A. muciniphila* growth.

BHI basal medium was supplemented with 0.05% (w/v) cysteine hydrochloride, and sodium butyrate was added at gradient concentrations of 0, 250, 500, 750, and 1000 μg/mL. The pH was adjusted to approximately 7.0. *A. muciniphila* was inoculated into each medium at a final density of 1 × 10^7^ CFU/mL and cultured at 37°C for 40 h.

The CLB cell lysate was prepared. CLB was cultured in RCM medium at 37°C for 20 h, harvested by centrifugation (6,000 × *g*, 10 min, 4°C), and washed three times with pre-chilled PBS to remove residual nutrients. The cells were then homogenized using a homogenizer, and the resulting lysate was filtered through a 0.22 μm membrane. The filtrate was subsequently added to BHI medium supplemented with 0.05% (w/v) cysteine hydrochloride. AKK (1×10^7^ CFU/mL) was inoculated into the lysate-supplemented BHI medium and incubated at 37°C for 48 h anaerobically.

The CLB culture supernatant was collected: CLB was cultured as above, centrifuged (6,000 × *g*, 10 min, 4°C), and the supernatant was pH-adjusted to 7.0 and filtered (0.22 μm). The supernatant was mixed with BHI medium at 25% (7.5 mL BHI + 2.5 mL supernatant) and 50% (5 mL BHI + 5 mL supernatant) ratios. Control groups included equivalent volumes of RCM medium mixed with BHI. AKK (1 × 10^7^ CFU/mL) was inoculated and incubated at 37°C for 48 h anaerobically.

In both experiments, bacterial growth was measured via optical density at 600 nm (OD_600_) using a microplate reader, with blank-adjusted values reported.

### Statistical Analyses

Student’s t-test was used for comparison between the control group and the HFD group. One-way ANOVA with post hoc Duncan multiple comparison tests was used for comparison among three groups. Wilcoxon rank-sum test was used to compare the Sobs and Chao among groups. Results were expressed in the mean ± SD unless otherwise indicated. *P* < 0.05 was considered statistically significant.

## Results

### Effects of CLB Intervention on Obesity-related Indexes of HFD-Mice

**Fig. 1:**
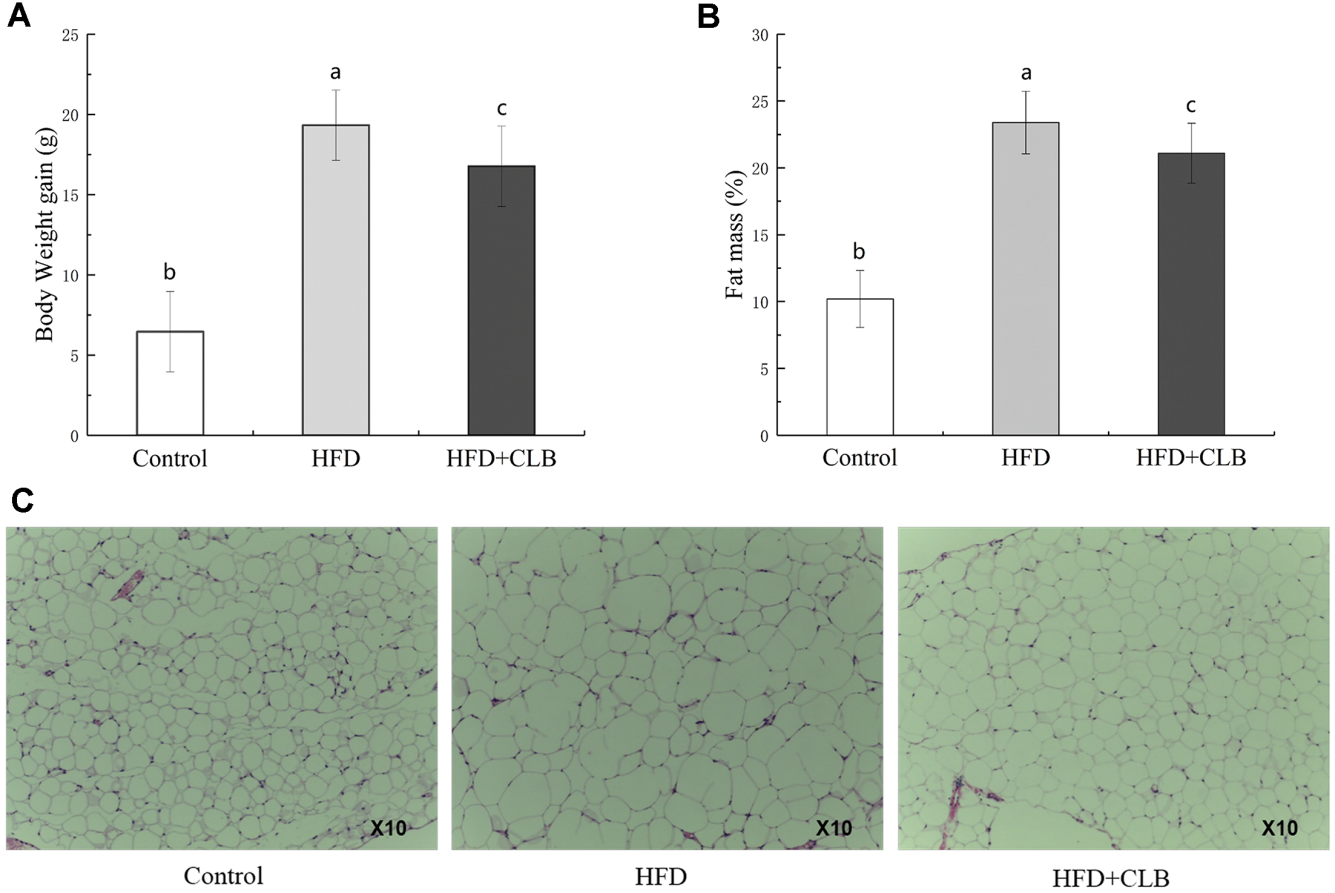
The obesity-related indexes of HFD-mice in different groups. (**A**) Body weight gain, (**B**) fat content, (**C**) adipose tissue size. Data are expressed as the means ± standard deviations. *P* < 0.05 compared the HFD with the Control group.

After 8 weeks of feeding, the body weight in the HFD group was over 15% higher than that in the control group, indicating the successful establishment of an obese mouse model. There was no significant difference in food intake between HFD and HFD + CLB groups (*P* > 0.05, Table S3). As shown in Fig. 1A, HFD group gained significantly higher body weight (19.32 ± 2.19 g) than the control group (6.45 ± 2.50 g), and CLB supplementation (HFD + CLB) reduced the body weight gain to 16.77 ± 2.23 g, representing a significant 13.20% reduction compared to the HFD group (*P* < 0.05). Similar results were observed for body fat mass (Fig. 1B). After 16 weeks of HFD administration, there was a significant increase in whole body fat mass (23.39 ± 2.34%) and adipose tissue weight compared to the control group. The group supplemented with *C. butyricum* (CLB) exhibited a lower fat mass (21.10 ± 2.24%) than the HFD group, although it remained significantly elevated compared to the control group, which had a fat mass of 10.20 ± 2.13%. As shown in Fig. 1C, the HFD group exhibited the largest adipocytes, whereas the HFD + CLB group showed smaller adipocytes than the HFD group. From these phenotypic indicators, we found that CLB intervention could alleviate the weight gain and fat mass caused by prolonged HFD feeding.

### Effects of CLB Intervention on the Short-Chain Fatty Acids in Feces

The control group exhibited significantly higher concentrations of acetate (288.62 ± 62.23 μg/g), propionate (129.26 ± 57.39 μg/g), and butyrate (63.25 ± 22.80 μg/g) compared to the HFD group. The HFD group showed substantially lower levels of these SCFAs. Supplementation with CLB markedly increased the concentration of butyrate, with its level rising to 11.73 ± 4.99 μg/g compared with the HFD group (*P* < 0.05). The results suggest that CLB may have a mitigating effect on the reduction of SCFA concentrations caused by a HFD, especially contributing to butyrate levels (Table 1).

### Effect of CLB Intervention on Intestinal Flora in Mice

**Fig. 2:**
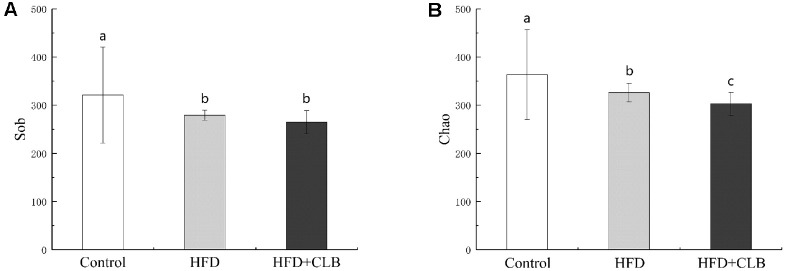
Alpha diversity analysis of gut microbiota in different groups. Rarefaction curve (**A**) and Shannon index curve (**B**) of all 31 samples. Different letters indicate significant differences between groups (*P* < 0.05).

**Fig. 3:**
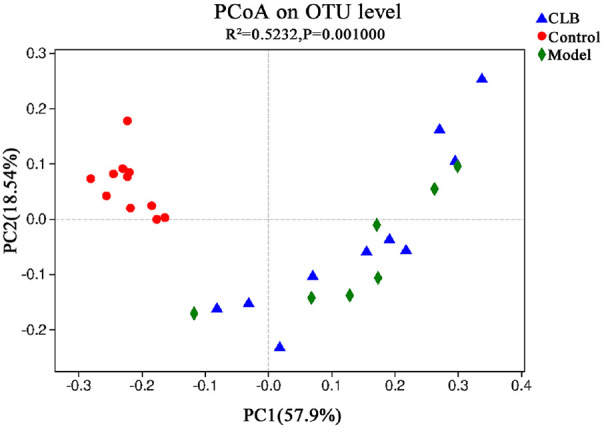
PCoA analysis of gut microbial community structures in different groups. PC1 (57.9%) and PC2 (18.54%) explain variance. PERMANOVA analysis (R^2^ = 0.5232, *P* = 0.001) indicated significant differences among groups. Model = HFD; CLB = HFD + CLB.

A total of 31 fecal samples from the Control, HFD, and HFD + CLB groups were analyzed for gut microbiota composition by 16S rRNA sequencing. Sobs index and Chao1 estimates were used to evaluate microbial diversity (Fig. 2A) and community richness (Fig. 2B), respectively. After HFD feeding, both Sobs and Chao1 indices significantly decreased compared with the Control group, indicating a decline in microbial richness. However, CLB intervention did not reverse the change of richness. According to the distance of the OTU level, the PCoA visually revealed that the microbiota structure of the control group clustered separately from both the HFD and HFD + CLB groups. PERMANOVA (R^2^ = 0.5232, *P* = 0.001) confirmed significant differences in microbial community composition among the three groups (Fig. 3), with 57.9% and 18.54% of the variation explained by PC1 and PC2, respectively. These results suggest that CLB had some impact on the richness or overall community structure of the HFD-shaped microbiota.

**Fig. 4:**
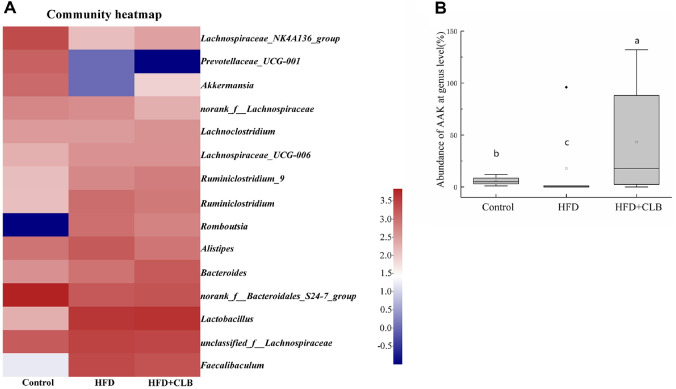
Modulation of gut microbiota and *Akkermansia muciniphila* by CLB supplementation. (**A**) Heatmap of the genus-level gut microbial community structure. (**B**) The relative abundance of *Akkermansia muciniphila* in intestinal microbiota in mice of each group. Different letters indicate significant differences between groups (*P* < 0.05).

Compared with the Control group, the abundances of 11 genera were significantly altered in the HFD group, which may be associated with obesity (Table S2 and Fig. 4A). *Lactobacillus* and *Alistipes* were enriched in the HFD group, while the abundance of *Bacteroidales*_S24-7_group, *Lachnospiraceae*_NK4A136_group, *Bacteroides*, *Prevotellaceae*_UCG-001, *Akkermansia*, *Alloprevotella*, *Ruminococcaceae*_UCG-014, *Prevotellaceae*_NK3B31_group and *Bifidobacterium* decreased in obesity group. Among these genera, the level of *Alistipes* and *Akkermansia* were significantly modulated by CLB intervention. As detailed, CLB decreased the high level of *Alistipes* induced by HFD, and CLB also significantly stimulated the abundance of *Akkermansia* which were restrained by obesity. The heatmap clearly showed that *Akkermansia* was markedly reduced in the HFD group compared with the Control group, but its abundance was restored following CLB supplementation in HFD-induced obese mice (Fig. 4A). The statistical analysis confirmed the same results (Fig. 4B). A total of three OTUs annotated as “*Akkermansia muciniphila* ATCC BAA-835” were detected in the fecal samples. Among them, one OTU was nearly absent (detected only in the Control group and thus excluded from downstream analysis), while the abundance of the other two OTUs in the CLB group was significantly higher than that in the HFD group (Fig. S1). But the heatmap did not show the differences between the three groups of *Alistipes*. These results suggest that the interaction between CLB and *A. muciniphila* may contribute to the anti-obesity effect of CLB.

### Effects of Butyrate, CLB Lysate, and Culture Supernatant on Growth of AKK *In Vitro*

**Fig. 5:**
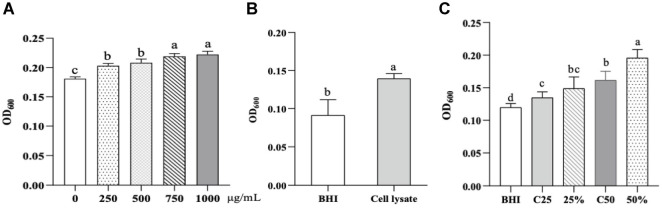
Effects of Butyrate and CLB - derived components on AKK growth. (**A**) Dose-dependent effects of sodium butyrate on the growth of AKK, assessed by OD_600_. (**B**) Growth of AKK in BHI medium or BHI medium supplemented with CLB cell lysate, measured by OD_600_. (**C**) Dose-dependent effects of CLB culture supernatant on AKK proliferation, evaluated by OD_600_. Data are expressed as the means ± standard deviations. Different letters indicate significant differences between groups (*P* < 0.05).

Butyrate and CLB-derived components significantly stimulated the growth of *A. muciniphila*. With increasing concentrations of butyrate in the culture system, the OD_600_ values of AKK gradually increased (Fig. 5A). In the cell lysate experiment, AKK cultured in BHI medium supplemented with CLB lysate exhibited a markedly higher OD_600_ than the lysate-free control (*P* < 0.05; Fig. 5B). Similarly, CLB culture supernatant demonstrated a dose-dependent enhancement of AKK proliferation. At 25% supernatant concentrations (25%) and 50% supernatant concentrations (50%), OD_600_ values were significantly elevated relative to both BHI-only and RCM-substituted controls (C25 and C50 respectively), with the 50% group achieving the highest growth promotion (Fig. 5C). These findings suggest that CLB lysate and supernatant contain bioactive metabolites or nutrients that directly promote the growth of *A. muciniphila*, highlighting potential synergistic interactions between the two bacterial species.

## Discussion

In this study, supplementing CLB could alleviate obesity in C57BL/6 mice induced by a HFD. Not unexpectedly, CLB increased the content of butyrate in the gut. More importantly, intervention of CLB could regulate certain genera involving obesity alleviation rather than restore the diversity of intestinal flora, by 16S rRNA gene sequencing and correlation analysis of intestinal microorganisms. *In vitro* culture experiments have confirmed that *C. butyricum* could significantly promote the proliferation of AKK bacteria. To our knowledge, this study provides the compelling evidence *in vitro* that butyrate and *C. butyricum* can promote the proliferation of AKK, suggesting a potential mechanistic explanation for its ability to modulate the gut microbiota and ameliorate obesity in mice.

Intestinal microbiota is increasingly thought to be involved in obesity, metabolic syndrome, and inflammation [23-25]. Numerous studies have confirmed that probiotics or prebiotics can regulate the intestinal microbiota, strengthen the intestinal barrier, and have the effect of improving obesity [26-28]. Probiotics can regulate the intestinal microbiota by inhibiting Gram-negative bacteria, reduce intestinal LPS, increase intestinal SCFAs levels [29-32], especially butyric acid, which can strengthen tight junction proteins, increase the intestinal epithelial barrier, and reduce LPS entry into the blood, thereby alleviating chronic inflammation caused by LPS and improving obesity [33-36]. Therefore, butyric acid producing bacteria are considered as microorganisms to improve obesity. *C. butyricum* is a spore-forming butyric acid-producing bacterium widely found in soil, healthy animals and human intestines [37-39]. Butyrate is one of the dominant fermentation end-products of *C. butyricum* via the butyrate kinase (buk) pathway. In addition, *C. butyricum* (CLB) also produces acetate, lactate, and other metabolites during its metabolic processes [40]. Under certain conditions, CLB can utilize acetate and lactate present in the system to synthesize butyrate [41, 42]. In this study, not surprisingly, feeding CLB partially reversed the HFD-induced low butyrate in feces (Table 1). However, the reduction in acetate levels was not alleviated but instead further decreased, which is likely attributable to the utilization of acetate - either self-produced or derived from other microbiota—by CLB for butyrate synthesis. Butyric acid was an energy source for epithelial cells, which was absorbed into cells by non-ionic diffusion or sodium-coupled monocarboxylate transporter (SMCT1 (SLC5A8)) coupled with Na^+^ concentration gradients. Butyric acid could promote the proliferation of intestinal epithelial cells and enhance the intestinal barrier [36, 43]. The above process can improve intestinal leakage and reduce the level of inflammation in the body, so as to improve abnormal lipid metabolism and insulin resistance. On the cell surface, butyrate acts as a ligand for G protein-coupled receptors (GPCR) (GPR43, GPR41 and GPR109) on intestinal epithelial cells [44]. GPCR activated by butyrate as a signal molecule in the intestine also produces the endocrine hormones glucagon-like peptide 1 (GLP-1) and peptide YY (PYY), preventing obesity and insulin resistance induced by a HFD [45]. Therefore, regulating intestinal butyric acid level is one of the mechanisms for CLB to improve obesity in mice.

We also found that CLB did not restore the variation of microbial diversity or overall profile induced by HFD. But CLB intervention recovered the specific genera. By comparing the changes in flora between each two groups, a significant difference was screened, and it was found that CLB can significantly improve the reduction of the *Akkermansia*’s abundances in the intestine due to HFD induction. *A. muciniphila* is a strict anaerobic bacterium in the gut. It was first isolated from human fecal samples in 2004 and has received much attention due to its negative association with obesity, diabetes, and inflammatory bowel disease [46]. AKK colonizes the mucous layer of the human gut and specifically degrades mucin to produce oligosaccharides and short-chain fatty acids. At the same time, mucin degradation stimulates the body to produce more mucin, which thickens the mucus layer and prevents LPS from entering the bloodstream from the intestine [47]. This is one of the mechanisms of anti-obesity of AKK in the intestine. Amuc_1100, a key outer membrane protein derived from AKK, is considered an important component which significantly contributes to obesity amelioration by modulating host immune responses and enhancing intestinal barrier function [48]. Specifically, it downregulates pro-inflammatory cytokines (*e.g.*, *TNF-α*, *IL-1β*, and *IL-6*) while upregulating anti-inflammatory *IL-10* and tight junction proteins (*e.g.*, ZO-1 and OCLN), thereby reducing systemic inflammation and metabolic endotoxemia associated with obesity [49, 50]. AKK's other metabolites, like butyrate, also regulate many transcription factors and gene expression during cell lipid metabolism and growth, such as fasting-induced adipose factor (Fiaf), histone deacetylase and peroxisome proliferator-activated receptor gamma (PPARγ) [51].

In this study, the abundance of *Akkermansia* in HFD group decreased, while CLB could significantly reverse low *Akkermansia* induced by HFD. This also indicates that there is a negative correlation between *Akkermansia* and obesity, and the increase in butyrate content is likely to be related to the abundance of AKK. Similar results were also observed by Pan [39], in which CLB increased AKK in mice with acute pancreatitis. There are two possible mechanisms underlying CLB's potential for increasing AKK abundance. On the one hand, CLB production of butyrate provides energy to intestinal epithelial cells, increases goblet cell activity [52, 53], and then increases mucin secretion, thereby increasing AKK abundance. On the other hand, butyrate may directly stimulate the growth of AKK [54, 55]. Using *in vitro* culture experiments, we have confirmed that butyrate and the supernatant from CLB cultures can directly stimulate the proliferation of AKK bacteria. We hypothesize that the butyrate produced by CLB metabolism may serve as an effective energy and carbon source, thereby promoting the proliferation of AKK bacteria.

Furthermore, it is important to acknowledge that the anti-obesity effects observed with CLB supplementation are likely multifactorial. While our data highlight a novel axis involving the direct promotion of AKK growth, the concurrently elevated butyrate levels derived from CLB undoubtedly contribute independently to metabolic improvement. Other studies have indicated that the regulatory effects of CLB in the gut are not mediated by a single metabolite or strain alone, but rather through complex interactions between its metabolic products and the gut microbiota [56]. Therefore, the alleviation of obesity by CLB probably results from a synergistic combination of microbial cross-feeding (promoting AKK) and the host-directed effects of its metabolic end-product. The mechanisms underlying these metabolite–microbiota interactions, including microbial cross-feeding, remain an important focus for our future research.

In conclusion, CLB supplementation in our study significantly reduced fat accumulation, improved obesity, and increased intestinal butyrate content in HFD-induced mice. CLB restored specific bacterial genera impaired by HFD rather than changed overall microbial diversity or community. It was observed that CLB significantly alleviated the decrease of *Akkermansia* caused by obesity in mice model. Interestingly, Butyrate and CLB supernatant promoted the growth of AKK directly. It is speculated that AKK may be one of CLB's intestinal targets for improving obesity, and provide a new perspective for further research on microbiota and obesity.

## Supplemental Materials

Supplementary data for this paper are available on-line only at http://jmb.or.kr.



## Figures and Tables

**Table 1:** The SCFAs content in the fecal samples.

Groups	Acetate	Propionate	Butyrate
Control	288.62 ± 122.32	129.26 ± 57.39	63.25 ± 22.80
HFD	23.61 ± 5.03	14.00 ± 5.71	7.27 ± 3.40
HFD + CLB	17.96 ± 2.44	7.25 ± 4.05	11.73 ± 4.99

Values are represented as the means ± SD. (μg/g; *P* < 0.05) as determined by Wilcoxon rank-sum test.
