# Supplementary material for: Oral Administration of Clostridium butyricum Alleviates High-Fat Diet-Induced Obesity in Mice by Modulating Gut Akkermansia muciniphila Abundance via Direct Growth Promotion
Source: J Microbiol Biotechnol. 2025 Dec 9;35:e2509005. doi: 10.4014/jmb.2509.09005 (PMC12706150; doi:10.4014/jmb.2509.09005)
Supplement: Supplementary file 1 [file jmb-35-e2509005-supple.zip › jmb-35-e2509005-supple1.pdf]

J. Microbiol. Biotechnol. 2025. 35: e2509005
https://doi.org/10.4014/jmb.2509.09005

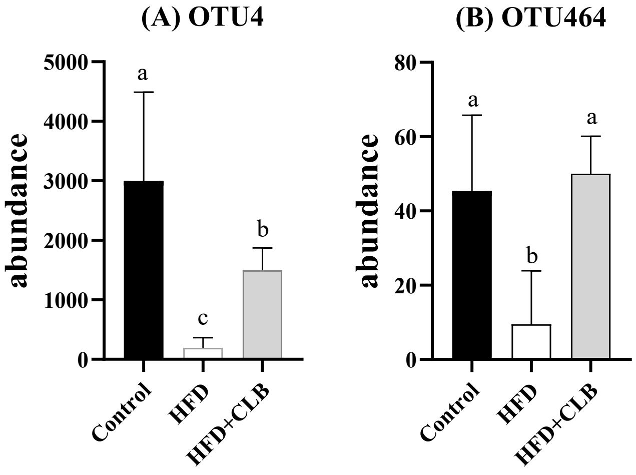

Fig. S1. Abundance of *Akkermansia* OTUs in different treatment groups. The abundance of two *Akkermansia* OTUs (OTU4 and OTU464) was measured in the Control, High-Fat Diet (HFD), and HFD + CLB treatment groups. Panel (A) shows the data for OTU4, with significant differences between the groups indicated by different letters (a, b, c), while panel (B) presents the data for OTU464. CLB intervention significantly restored the abundance of both OTUs, which had been reduced by the HFD treatment. Data are presented as mean ± standard deviation.
